# TiHo-0906: a new feline mammary cancer cell line with molecular, morphological, and immunocytological characteristics of epithelial to mesenchymal transition

**DOI:** 10.1038/s41598-018-31682-1

**Published:** 2018-09-05

**Authors:** José Luis Granados-Soler, Johannes Junginger, Marion Hewicker-Trautwein, Kirsten Bornemann-Kolatzki, Julia Beck, Bertram Brenig, Daniela Betz, Jan Torben Schille, Hugo Murua Escobar, Ingo Nolte

**Affiliations:** 1 Small Animal Clinic, University of Veterinary Medicine Hannover Foundation, Hannover, Germany; 2 Department of Pathology, University of Veterinary Medicine Hannover Foundation, Hannover, Germany; 3 Chronix Biomedical, Göttingen, Germany; 4 Institute of Veterinary Medicine, University of Göttingen, Göttingen, Germany; 5 Department of Internal Medicine, Medical Clinic III, Clinic for Haematology, Oncology and Palliative Care, University Medical Center Rostock, Rostock, Germany

## Abstract

Feline mammary carcinomas (FMCs) with anaplastic and malignant spindle cells histologically resemble the human metaplastic breast carcinoma (hMBC), spindle-cell subtype. hMBCs display epithelial-to-mesenchymal transition (EMT) characteristics. Herein we report the establishment and characterization of a cell line (TiHoCMglAdcar0906; TiHo-0906) exhibiting EMT-like properties derived from an FMC with anaplastic and malignant spindle cells. Copy-number variations (CNVs) by next-generation sequencing and immunohistochemical characteristics of the cell line and the tumour were compared. The absolute qPCR expression of EMT-related markers *HMGA2* and *CD44* was determined. The growth, migration, and sensitivity to doxorubicin were assessed. TiHo-0906 CNVs affect several genomic regions harbouring known EMT-, breast cancer-, and hMBCs-associated genes as *AKT1*, *GATA3*, *CCND2*, *CDK4*, *ZEB1*, *KRAS*, *HMGA2*, *ESRP1*, *MTDH*, *YWHAZ*, and *MYC*. Most of them were located in amplified regions of feline chromosomes (FCAs) B4 and F2. TiHo-0906 cells displayed an epithelial/mesenchymal phenotype, and high *HMGA2* and *CD44* expression. Growth and migration remained comparable during subculturing. Low-passaged cells were two-fold more resistant to doxorubicin than high-passaged cells (IC50: 99.97 nM, and 41.22 nM, respectively). The TiHo-0906 cell line was derived from a poorly differentiated cellular subpopulation of the tumour consistently displaying EMT traits. The cell line presents excellent opportunities for studying EMT on FMCs.

## Introduction

Feline mammary tumours are the third most common neoplasms in female cats^1^. The feline mammary tissue encompasses three tissue lineages, the luminal epithelial, the myoepithelial, and the mesenchymal^2^. Around 90% of feline mammary neoplasms are luminal epithelial tumours usually referred to as FMCs^2,3^. FMCs are invasive tumours characterized by early metastasis^3,4^. FMCs with anaplastic and malignant spindle cells are uncommon, and their distinctive morphologic features are not described in any of the subtypes included in the latest classification published by the World Health Organization (WHO)^2^. The genetic determinants of the neoplastic spindle-cell component in FMCs are still unclear, and little is known about the biological behavior of these tumours and prognosis of the affected animals.

FMCs with malignant anaplastic and spindle cells share some histological characteristics with the highly malignant hMBCs, spindle-cell subtype. Histologically, hMBCs display epithelial differentiation towards mesenchymal elements (chondroid, osseous, rhabdoid, and spindle)^5^. Around 80% of hMBCs are spindle-cell tumours frequently enriched in EMT features^6–8^. EMT is an embryonic process reactivated in adult tissues during cicatrization, fibrosis, and cancer^9^. During EMT, epithelial cells lose expression of cell-cell junction proteins and gain the expression of mesenchymal proteins^10^. Afterwards, the EMT-derived cells secrete proteolytic enzymes (metalloproteinases), which degrade the extracellular matrix and cell-cell junctions, facilitating detachment, mobility, and metastasis^11^. EMT results in enhanced migratory capacity^7,10^, cancer stem cells (CSCs) properties^9,12–15^, and drug resistance^9,16,17^. Usually, neoplastic cells do not experience a full EMT, instead, they assume different phenotypes along the epithelial-mesenchymal axis^17–20^.

The EMT is regulated by several cytokines and growth factors^9,15,21^. Consequently, it has been induced in cell culture by different methods^13,14,22,23^. The High-mobility group AT-hook 2 protein (HMGA2) activates a range of EMT transcription factors implicated in the repression of epithelial genes, and mesenchymal genes up-regulation^24,25^. Therefore, EMT-derived cells are usually characterized by a higher HMGA2 expression^9,26^, loss or reduced expression of E-cadherin (E-cad), up-regulation of vimentin (Vim)^7,9^, and co-expression of epithelial markers (cytokeratins [CKs]) and mesenchymal markers (calponin [CALP], smooth muscle actin [SMA], and Vim)^7,27^. Other markers like CD44 participate in the downregulation of E-cad^12,28^. Consequently, EMT-derived cells are characterized by a higher CD44 expression^8,28^. The overexpression of this cell surface protein leads to enhanced cell migration, cancer invasion and metastasis^28^. Additionally, a higher CD44 expression in combination with other surface markers is used for CSCs identification^29,30^.

CNVs (copy-number gains [CNGs], and copy-number losses [CNLs]) are structural aberrations usually affecting extensive regions of the genome^31^. CNVs affect the gene expression patterns by altering the gene dosage in human breast cancer^32–34^, hMBCs^7,32^, and breast cancer cell lines^35^. Additionally, specific CNGs are concordant with EMT-related genes up-regulation in multiple human cancer types^36^. Cancer cell lines are characterized by genomic instability and structural dynamism^37–39^, which makes CNVs a suitable tool to understand cancer cell adaptation to the *in vitro* environment during cell culture establishment and clonal selection during subculturing. To the extent of our knowledge, this is the first study characterizing CNVs in an FMC cell line. This study reports on the establishment and comprehensive characterization of a cell line TiHo-0906 derived from a poorly differentiated FMC with anaplastic and spindle cells. The original tumour and the derived cell line were enriched with EMT-associated traits.

## Results

### Histopathological description of the tumour

**Figure 1:**
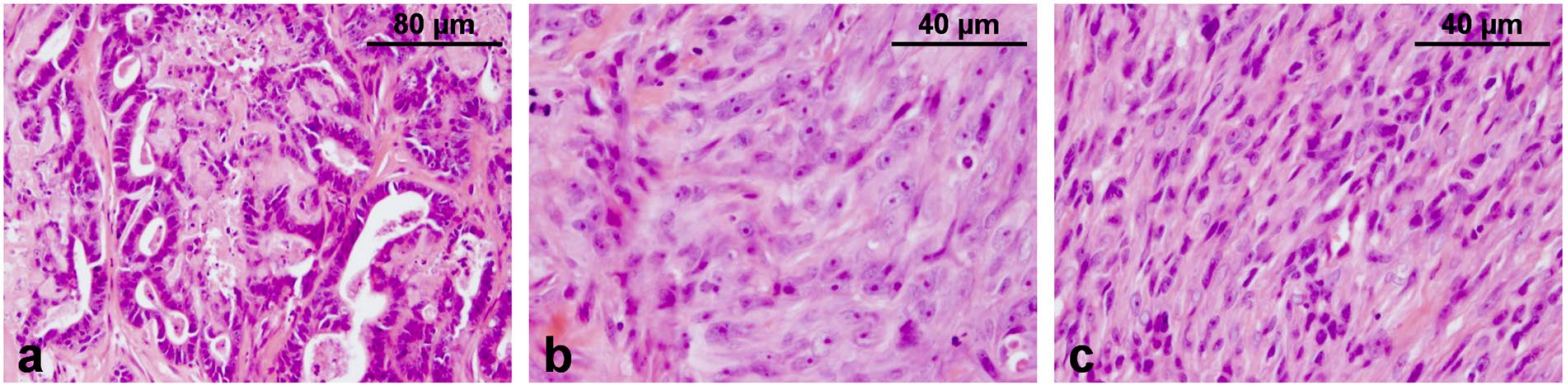
Histopathology. Tumour paraffin sections, H&E. The neoplasm, of which the cell line TiHo-0906 is derived, was characterized by (**a**) tubular, (**b**) solid anaplastic, and (**c**) spindle areas.

Histologically, the tumour was mainly composed of malignant spindle cells, malignant tubular epithelial cells, and small areas with anaplastic polygonal cells. The mammary gland was infiltrated by a multinodular invasive growing neoplasm. Some areas were characterized by cuboidal to columnar epithelial cells that formed irregular tubular structures by palisading along a basement membrane (Fig. 1a). These cells showed distinct cytoplasmic borders, moderate amounts of eosinophilic cytoplasm and central round basophilic nuclei with finely stippled chromatin and distinct nucleoli. In addition, there were multifocal areas in which neoplastic cells were arranged in solid nests of variable size with anaplastic morphology lacking tubular differentiation (Fig. 1b). Cells in these areas were featured by a polygonal shape with mostly well-defined cell borders and low to moderate amounts of eosinophilic cytoplasm. Their large prominent centrally arranged basophilic round to oval nuclei revealed a finely to coarsely stippled chromatin pattern and distinct prominent nucleoli. In contrast, some areas were dominated by closely packed spindle cells with low amounts of cytoplasm and oval to elongated basophilic nuclei containing finely stippled chromatin and indistinct nucleoli (Fig. 1c). Predominating within the anaplastic foci, the cells were featured by moderate anisokaryosis and anisocytosis and occasionally contained multiple nuclei. Mitotic figures averaged three per high power field. Clusters of tumour cells were also present within some lymphatic vessels. Multifocally, there were single cells or coalescing necrosis characterized by hypereosinophilia and nuclear pyknosis, rhexis and lysis. Neoplastic cells were supported by low to moderate amounts of collagen-rich fibrovascular stroma that showed multifocal infiltration by moderate numbers of lymphocytes and plasma cells as well as variable numbers of neutrophils.

### Cytomorphologic features of the cell line

**Figure 2:**
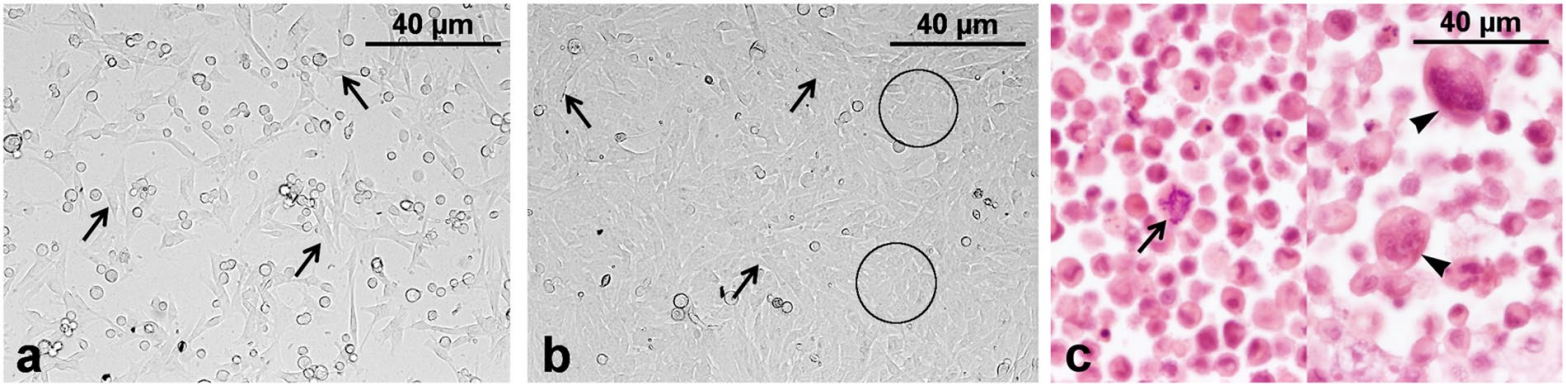
Cellular morphology. (**a**) TiHo-0906 P76 cell culture at subconfluence, inverted microscopy. Bipolar to multipolar shaped cells (arrows). (**b**) TiHo-0906 P76 cell culture at confluence, inverted microscopy. Monolayer of epithelial-like cells characterized by polygonal (circles) to bipolar morphology (arrows). (**c**) TiHo-0906 P79 pellet; paraffin sections, H&E. Round to polygonal shaped cells characterised by marked anisokaryosis and anisocytosis, atypical mitotic figure (arrow) and large multinucleated syncytia (arrowheads).

After cell culture establishment, TiHo-0906 cells proliferated in a monolayer. Subconfluent cells were spindle-shaped with a bipolar to multipolar morphology (Fig. 2a). At confluence, cells revealed a more epithelioid morphology with polygonal to bipolar shape (Fig. 2b). Cellular morphology and proliferation rate remained stable during prolonged subculturing (over 100 passages). Microscopic examination of TiHo-0906 cells in H&E stained paraffin sections from pellets revealed round to polygonal cells and a moderate to partly abundant cytoplasm. They had large, prominent, round to oval nuclei that showed moderate to severe atypia and contained coarsely stippled to vesiculated chromatin and distinct nucleoli. TiHo-0906 cells were further characterized by marked anisokaryosis and anisocytosis, atypical mitotic figures and several large multinucleated syncytia (Fig. 2c).

### Tumour immunophenotyping

Tumours displaying EMT features usually express epithelial and mesenchymal markers concurrently^7^. In general, a co-expression of epithelial markers and Vim was observed in tubular and solid anaplastic areas of the tumour. In contrast, spindle cells were negative to all epithelial markers and positive to all mesenchymal markers used. Additionally, all parts of the tumour were negative to the specific myoepithelial markers cytokeratin 5/6 (CK5/6) and p63 (Table 1).

**Table 1:** Comparative expression profile of the original tumour.

Marker	Tubuli-forming cells	Polygonal cells	Spindle cells
E-cad	+	−	−
CK8/18	+	+	−
pan-CK	+	+	−
CK14	+	+	−
CK5/6	−	−	−
p63	−	−	−
SMA	−	+	+
CALP	−	+	+
Vim	+	+	+
CD44	+	+	+

^+^Positive, −Negative.

**Figure 3:**
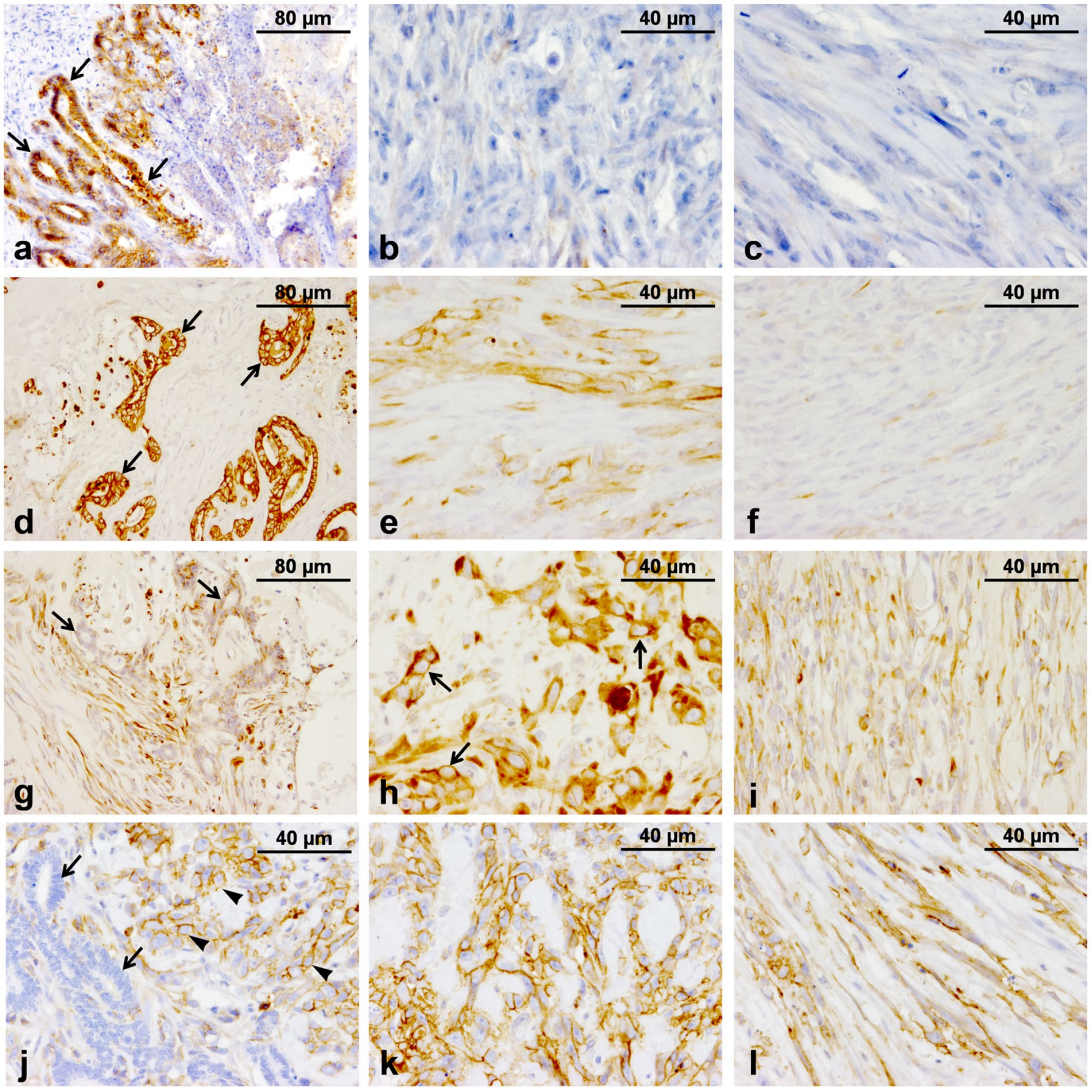
Immunohistochemical characteristics of the tumour. (**a**) E-cad; tubular epithelial cells strongly positive (arrows). (**b**) E-cad; polygonal cells in solid anaplastic areas of the tumour are negative. (**c**) E-cad; well-differentiated spindle cells negative. (**d**) pan-CK; intense cytoplasmic immunostaining by the neoplastic tubular cells (arrows). (**e**) pan-CK; polygonal cells moderately positive. (**f**) pan-CK; spindle cells negative. (**g**) Vim; moderate immunolabeling of neoplastic tubular epithelial cells (arrows). (**h**) Vim; intense cytoplasmic immunolabeling of the polygonal cells. (**i**) Vim; spindle cells moderately positive. (**j**) CD44; neoplastic tubular cells negative (arrows), polygonal cells moderately to intensely positive (arrowheads). (**k**) CD44; moderate to intense membrane staining of polygonal cells. (**l**) CD44; spindle cells moderately positive.

Tubular epithelial cells were strongly immmunoreactive to the luminal epithelial marker E-cad (Fig. 3a), while polygonal and spindle cells were negative (Fig. 3b,c). Tubular epithelial cells and polygonal cells were positive for the general epithelial marker pan-cytokeratin [pan-CK] while spindle cells were negative (Fig. 3d–f). A positive reaction to Vim was observed in all areas of the tumour (Fig. 3g–i).

HMGA2 elicits EMT by activating a series of transcription factors^24,25^, while CD44 participates in the downregulation of E-cad commonly observed during EMT^12,28^. Immunohistochemically, there were no specific cross-reactivity of the HMGA2 selected antibody with feline tissue. On the other hand, tubular epithelial cells were negative for CD44 (Fig. 3j), while numerous CD44-positive polygonal and spindle tumour cells were observed (Fig. 3k,l).

Tubular epithelial cells were slightly positive to oestrogen and progesterone receptors (ER and PR) and negative to the human epidermal receptor-2 (HER-2). Polygonal cells were negative to ER and PR, but slightly positive to HER-2. Whereas, well-differentiated spindle cells were negative for all hormonal markers evaluated. The role of cyclooxigenase-2 (COX-2), p53, claudin 2 (CLDN-2), and ki67 as indicators of tumor aggressiveness and prognosis has been documented in FMCs^40–44^. Moreover, loss or reduced expression of claudins, in general, may contribute to the development of EMT^45–47^. In this case, the tumour was negative to COX-2. The expression of p53 was weak in tubular and polygonal cells, whereas well-differentiated spindle cells were negative. CLDN-2 was slightly positive in tubular and polygonal cells; spindle cells were negative. Additionally, a high Ki-67 proliferation index was observed in all areas of the tumour^44^. Results of the immunohistochemical characteristics of the tumour are detailed in Table 2.

**Table 2:** Immunohistochemical characteristics of the tumour.

Marker	Tubuli-forming cells	Polygonal cells	Spindle cells
ER	17.5%	0	0
PR	27.6%	0	0
HER-2^**^	0	1+	0
COX-2^*^	0	0	0
p53	6.3%	3.3%	0
CLDN-2^***^	8	6	1
Ki67	47.3%	24.5%	22.4%

*COX-2 scoring system^93^, **HercepTest^94,95^, ***CLDN2 scoring system^42^.

### Cell line phenotyping

TiHo-0906 cells at low and high passage reacted similarly to the markers employed; all pellets evaluated coexpressed epithelial and mesenchymal markers (Table 3).

**Table 3:** Comparative expression profile of the cell line.

Marker	TiHo-0906 early passage			TiHo-0906 high passage		
	pellet 1	pellet 2	pellet 3	pellet 1	pellet 2	pellet 3
E-cad	+	+	+	+	+	+
CK8/18	+	+	+	+	+	+
pan-CK	+	+	+	+	+	+
CK14	+	+	+	+	+	+
CK5/6	−	−	−	−	−	−
p63	−	−	+	+	+	−
SMA	+	+	+	+	+	+
CALP	+	+	+	+	+	+
Vim	+	+	+	+	+	+
CD44	+	+	+	+	+	+

+Positive, −Negative.

**Figure 4:**
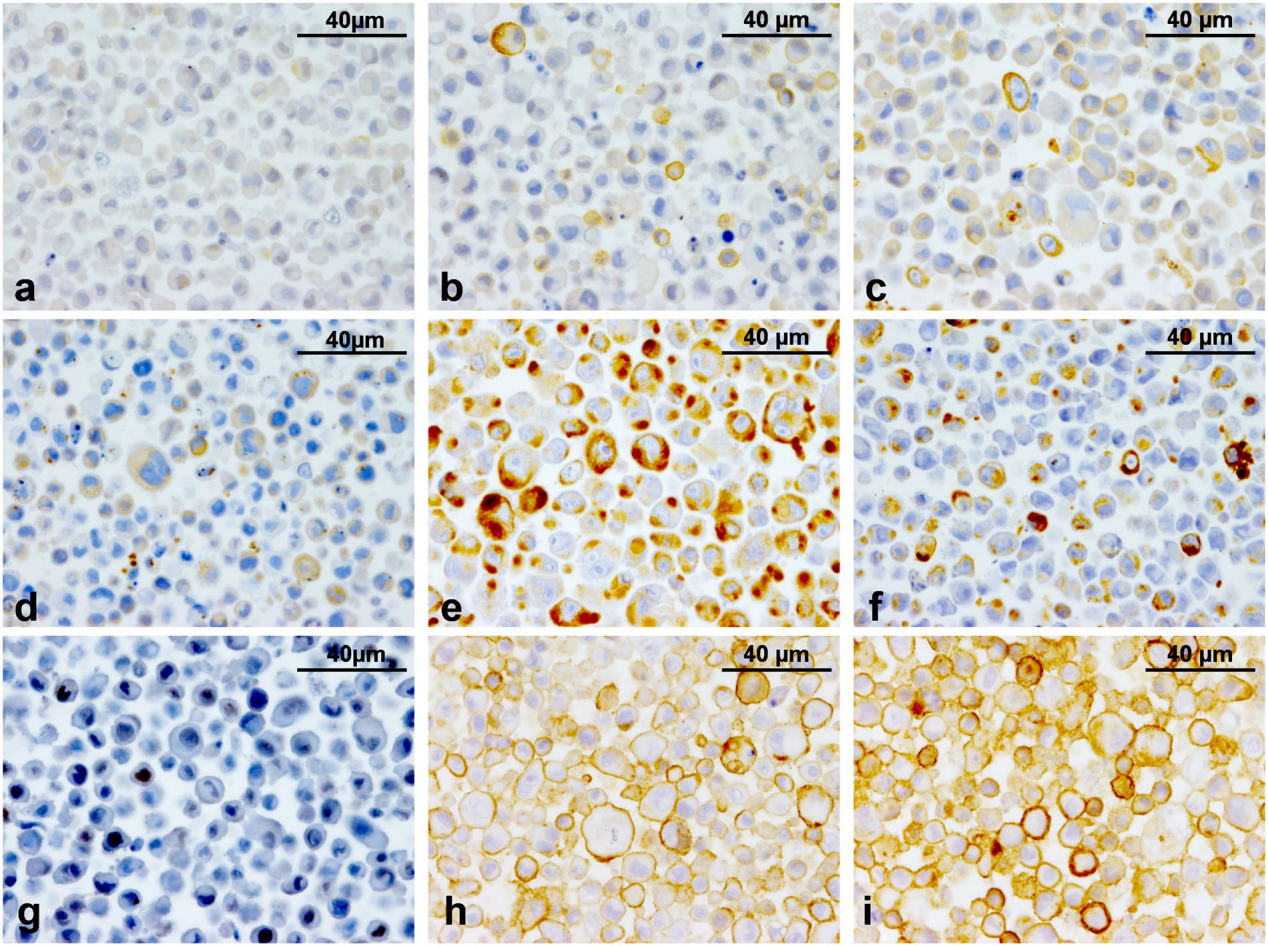
Immunohistochemical characteristics of the TiHo-0906 cell line. (**a**) E-cad, low passage (P8); most cells display a weak cytoplasmic and membranous labelling. (**b**) E-cad, high passage (P80); weak cytoplasmic and membranous labelling in most cells, some cells display a more intense reaction. (**c**) pan-CK, low passage (P8); moderate cytoplasmic labelling, some cells display a more intense reaction. (**d**) pan-CK, high passage (P80); moderate cytoplasmic labeling. (**e**) Vim, low passage (P8); strong cytoplasmic labelling and numerous positive cells. (**f**) Vim, high passage (P80); most of the cells are moderately positive, some cells are strongly positive. (**g**) p63, low passage (P8); moderate nuclear labelling in most of the cells, some nuclei are strongly positive. (**h**) CD44, low passage (P8); cellular membranes are moderately positive. (**i**) CD44, high passage (P80); moderate membranous labelling in most of the cells, some cells are strongly positive.

The immunohistochemical characteristics of the cell line resembled those of the polygonal epithelial cells present in the anaplastic areas of the tumour with exception of E-cad that was positive in most TiHo-0906 cells at low (Fig. 4a) and high passages (Fig. 4b). TiHo-0906 cells at low (Fig. 4c) and high passages (Fig. 4d) were positive for pan-CK, and Vim (Fig. 4e,f). Interestingly, some of the pellets evaluated in both low- and high-passaged cells were positive for the myoepithelial marker p63 (Fig. 4g, low-passaged cells), while the tumour was negative. An intense membrane staining for CD44 was observed on almost 100% of the cells in all pellets evaluated (Fig. 4h,i, low and high passages respectively).

TiHo-0906 cells were slightly positive to the hormonal receptors (ER, PR), and HER-2. COX-2 labelling was negative in all of the pellets evaluated. p53 was moderately expressed in all of the pellets evaluated. CLDN-2 was weakly expressed in all pellets (score 1 to 3)^42^. The mean Ki-67 proliferation index of TiHo-0906 cells in pellets was 39.3% and 42.2% in early and high passages, respectively.

### Copy number variation analysis

**Figure 5:**
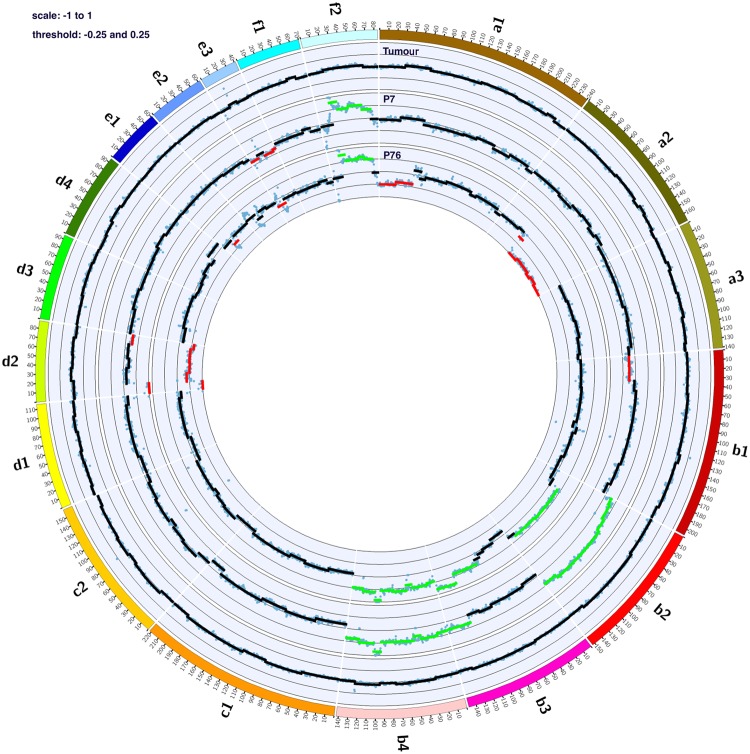
Comparative Circos plots of the original tumour and TiHo-0906 cells at low and high passages. Outer multicolor ring: chromosome location. Inner rings from the outside-in: original tumour, TiHo-0906 P7, and TiHo-0906 P76. Green lines indicate CNGs, and red lines indicate CNLs.

TiHo-0906 cells at low (P7) and high (P79) passages showed a high level of genomic instability when compared to the original tumour. The frequency of CNVs in TiHo-0906 cells increased during *in vitro* culturing. FCAs B2, B3, B4, and F2 in TiHo-0906 cells displayed most of the genomic regions with CNGs (Fig. 5).

CNGs in chromosomes B2, B4 and F2 remained similar during subculturing. Interestingly, chromosomes B4 and F2 harbour some of the previously reported EMT-implicated genes associated with more frequent CNGs in different types of human cancer^36^ (Table 4).

**Table 4:** EMT-associated genes frequently affected by CNGs in multiple types of human cancer and their copy number status in TiHo-0906 cells.

Gene	Feline chromosome	Location (bp)	TiHo-0906 P7	TiHo-0906 P76
**EMT-associated CNGs** ^36^				
*EGFR* ^*, **^	A2	69,024,076–69,243,754		↓
*TWIST1*	A2	113,452,909–113,454,730		↓
*AKT1* ^*^	B3	148,724,327–148,745,203	↑	↑
*VIM*	B4	16,166,246–16,173,638	↑	↑
*ZEB1*	B4	28,693,675–28,892,926	↑	↑
*KRAS*	B4	60,631,473–60,670,232	↑	↑
*ESRP1*	F2	43,419,796–43,484,114	↑	↑
*MTDH*	F2	46,121,204–46,179,417	↑	↑
*YWHAZ*	F2	49,027,370–49,061,094	↑	↑
*MYC* ^*, **, ****^	F2	70,514,069–70,519,003	↑	↑

^*^Breast cancer-associated CNGs^33,34^, ^**^hMBCs-associated CNGs^7,32^, ^****^Breast cancer cell lines-associated CNGs^35^.

In general, the amount of CNLs also increased during cultivation. High-passaged TiHo-0906 cells showed losses on proximal chromosome A1, distal chromosome A2 and a focal deletion on chromosome E1 that were not observed in low-passaged cells. However, low-passaged TiHo-0906 cells showed a focal deletion of proximal chromosome B1 that was not observed in high-passaged cells. Additionally, the size of CNLs observed in chromosome E3 decreased during sub-cultivation. Some of the most important breast cancer-related genes in humans are located in the analysed corresponding feline regions^7,32,33^ showing amplifications as well as deletions (details in Table 5).

**Table 5:** Breast cancer and specific hMBC-associated Genes frequently affected by CNVs and their copy number status in TiHo-0906 cells.

Gene	Feline chromosome	Location (bp)	TiHo-0906 P7	TiHo-0906 P76
**Breast cancer-associated CNGs** ^33, 34^				
*TERT*	A1	239,584,995–239,606,213		↓
*CCND3* ^**^	B2	42,899,534–42,997,754		↑
*FOXA1*	B3	86,949,330–86,955,050		↑
*MDM2*	B4	93,710,547–93,739,225		↑
*NF1*	E1	19,031,895–19,383,941		↓
*PPM1D*	E1	25,189,431–25,236,039		↓
*EIF3* ^****^	F2	55,394,876–55,449,634	↑	↑
*PKHD1L1* ^****^	F2	56,539,146–56,697,161	↑	↑
*CSMD3* ^****^	F2	59,042,991–60,300,161	↑	↑
*ZHX2* ^****^	F2	68,234,407–68,397,794	↑	↑
*SAMD12* ^****^	F2	64,396,137–64,785,172	↑	↑
*EXT1* ^****^	F2	64,046,201–64,334,375	↑	↑
*MRPL13* ^****^	F2	66,202,612–66,254,545	↑	↑
*MTBP* ^****^	F2	66,254,741–66,334,938	↑	↑
*SNTB1* ^****^	F2	66,342,257–66,585,108	↑	↑
*RNF139* ^****^	F2	69,585,566–69,597,426	↑	↑
*TATDN1* ^****^	F2	69,586,490–69,643,856	↑	↑
**Breast cancer-associated CNLs** ^33, 34^				
*BRCA2*	A1	11,562,333–11,623,186		↓
*RB1*	A1	22,863,380–23,038,325		↓
*MLL2/KMT2C*	A2	166,021,109–166,309,274		↓
*CSMD1* ^*****^	B1	2,777,407–4,810,740	↓	
*AGPAT5* ^*****^	B1	6,723,000–6,783,055	↓	
*TUSC3* ^*****^	B1	22,910,796–23,201,016	↓	
*DLC1* ^*****^	B1	25,013,341–25,575,894	↓	
*CLDN23* ^*****^	B1	26,341,593–26,342,694	↓	
*MFHAS1* ^*****^	B1	26,460,461–26,562,571	↓	
*FOXO3*	B2	98,320,907–98,445,633	↑	↑
*GATA3*	B4	7,378,473–7,400,352	↑	↑
*PTEN* ^*****^	D2	7,565,534–7,659,710	↓	↓
**hMBCs-associated CNGs** ^7, 32^				
*CCND2*	B4	39,524,203–39,554,520	↑	↑
*CDK4*	B4	86,164,754–86,167,722	↑	↑
**Additional important genes in regions displaying CNVs in TiHo-0906 cells**				
*FOXO1*	A1	18,849,598–18,947,131		↓
*AHR*	A2	111,731,783–111,787,715		↓
*MYB*	B2	124,810,249–124,844,570	↑	↑
*PFDN5*	B4	81,683,255–81,686,020	↑	↑
*HMGA2*	B4	93,165,641–93,304,557	↑	↑
*KSR1*	E1	18,893,366–19,057,414		↓
*RAC1*	E3	5,019,032–5,042,577	↓	↓

^**^hMBCs-associated CNGs^7,32^, ^***^hMBCs-associated CNLs^7,32^, ^****^Breast cancer cell lines-associated CNGs^35^, ^*****^Breast cancer cell lines-associated CNLs^35^.

### Real-time PCR expression analyses of *HMGA2* and *CD44*

**Figure 6:**
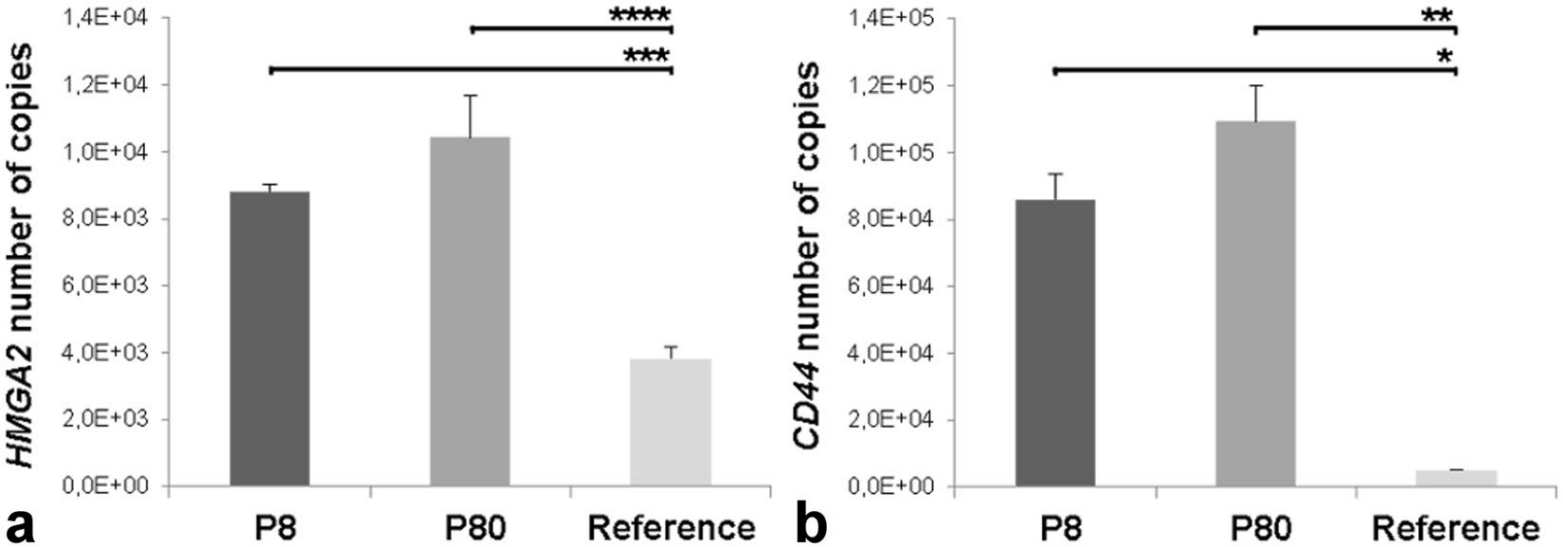
*HMGA2* and *CD44* qPCR-based expression. Comparative expression of (**a**) *HMGA2* and (**b**) *CD44* in TiHo-0906 cells at low (P8) and high (p80) passage versus selected reference tissues (feline testis, and healthy mammary feline tissue, respectively). Data are displayed as mean (SD); *p < 0.05, **p < 0.01, ***p < 0.001, and ****p < 0.0001.

The levels of *HMGA2* and *CD44* in TiHo-0906 cells were examined by absolute RT-PCR and compared to the corresponding reference tissues (feline testis, and healthy mammary feline tissue, respectively). The level of *HMGA2* expression in low and high passaged cells was higher than those of the reference tissue (*p* = 0.001, and *p* < 0.0001, respectively). Similarly, the *CD44* absolute expression in TiHo-0906 cells at low and high passages was significantly higher in comparison to the reference tissue (*p* = 0.01, and *p* = 0.0015, respectively). No significant differences in *HMGA2* and *CD44* expression were observed between low and high passages (Fig. 6).

### Growth behaviour and migration activity

**Figure 7:**
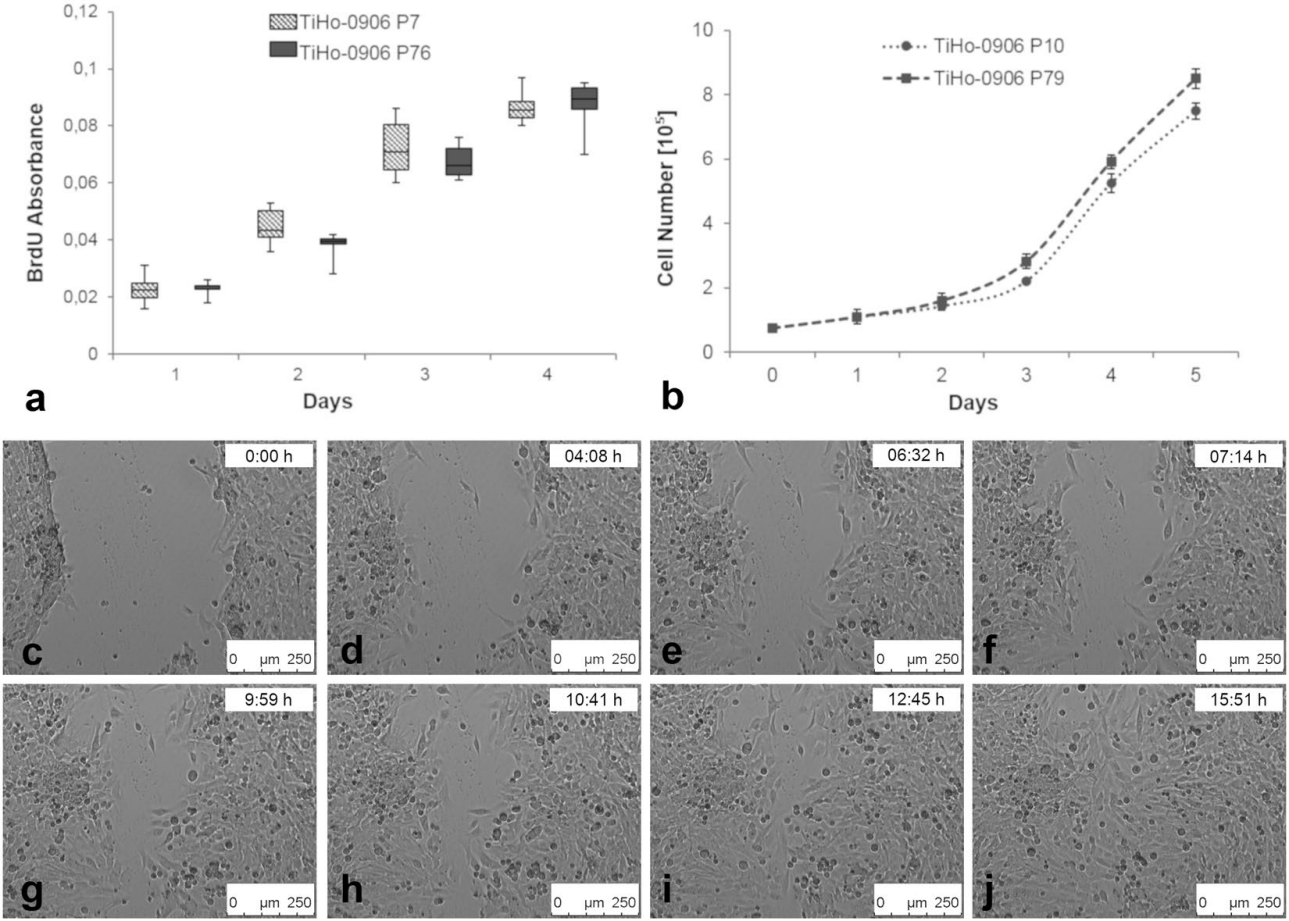
Cell proliferation, growth curves and *s*cratch assay. (**a**) BrdU cell proliferation assay of TiHo-0906 at low and high passages, absorbance values expressed as Max V [delta 370–492]. (**b**) Growth curves of TiHo-0906 at low and high passages, data are shown as mean (SD). (**c**–**j**) Scratch assay, TiHo-0906 P76 cell culture at inverted microscopy (10×).

No statistical differences in BrdU incorporation and growth curves were found between low and high passages of the TiHo-0906 cell line (Fig. 7a,b). The doubling time for the cell line was 28.9 h at low passage and 27.4 h at high passage. Fourteen hours after the scratching, migrating cells completely covered the wound (400 µm) in early and high passages of the TiHo-0906 cell line (Fig. 7c–j). No significant differences regarding the time to wound closure were observed between low and high passages.

### Metabolic activity of TiHo-0906 cells after doxorubicin treatment

**Figure 8:**
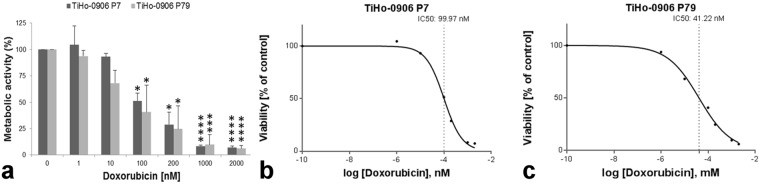
Influence of doxorubicin on metabolic activity of TiHo-0906 cells using MTS-test. (**a**) Doxorubicin resistance analysis of TiHo-0906 cells at low and high passage. Data are displayed as mean (SD) of metabolically active cells (%). Significance was calculated by comparing doxorubicin-treated versus untreated cells; *p < 0.05, **p < 0.01, ***p < 0.001, and ****p < 0.0001. Doxorubicin dose-response curves of (**b**) TiHo-0906 cells at low passage, IC50: 99.97 nM and (**c**) TiHo-0906 cells at high passage, IC50: 41.22 nM.

According to the MTS assay results, the metabolic activity of doxorubicin-treated TiHo-0906 cells at low and high passage starts to significantly decrease at 100 nM of doxorubicin (*p* = 0.01, and *p* = 0.03, respectively), for details see Fig. 8a. After incubation with different concentrations of doxorubicin, the metabolic activity of TiHo-0906 cells at low and high passages was not statistically different. However, the IC50 of cells at low passage was approximately 2-fold higher than the IC50 of cells at high passage (Fig. 8b,c).

### Effects of doxorubicin on apoptosis and cytotoxicity

**Figure 9:**
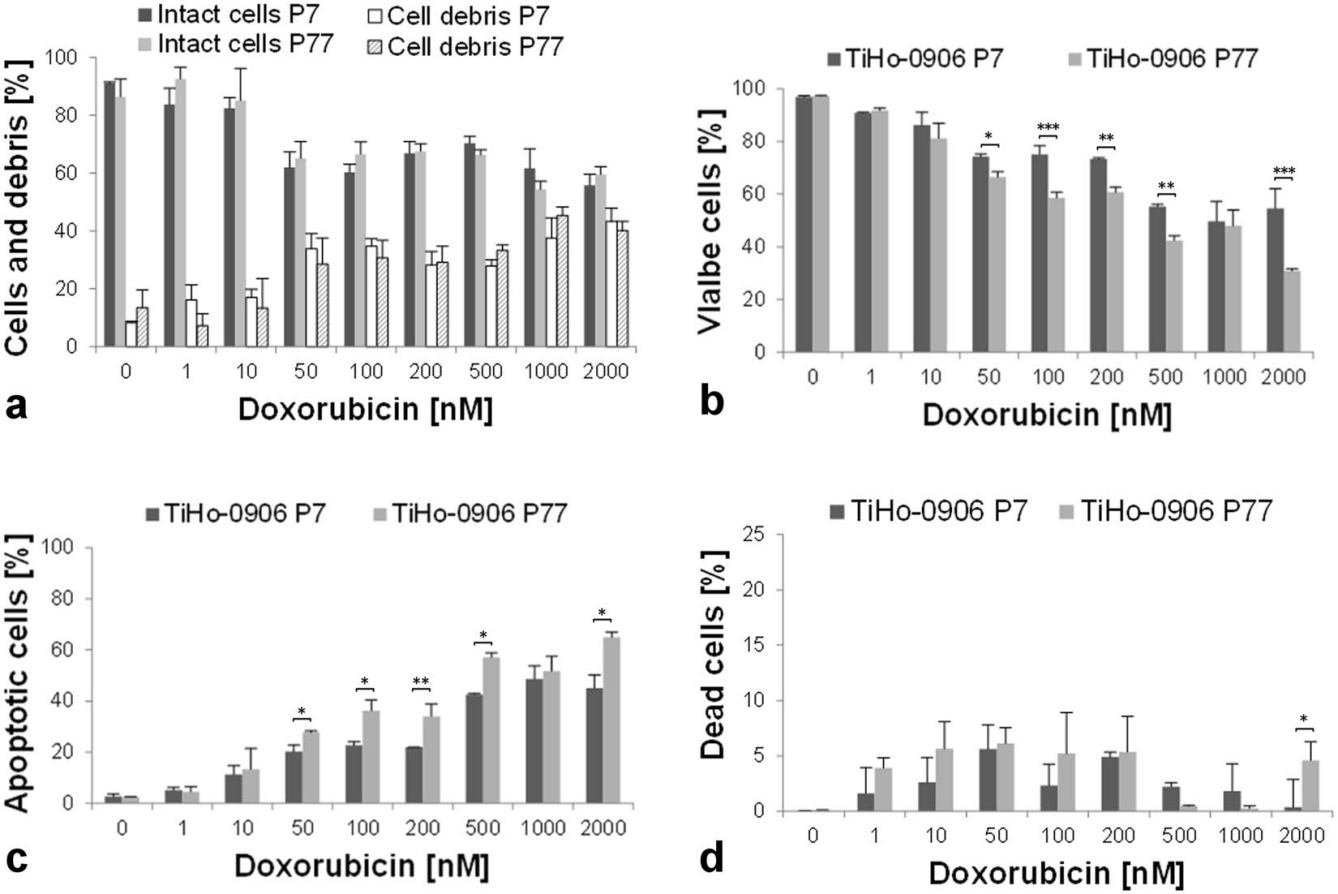
Flow cytometric assessment of doxorubicin effects on TiHo-0906 cells. (**a**) Percentage of intact cells (1^st^ and 2^nd^ column, low and high passage; respectively), and cell debris (3^rd^ and 4^th^ column, low and high passage; respectively) after incubation with doxorubicin. Cellular debris increases in proportion to the concentration of doxorubicin while intact cells decrease. Notice the higher amount of intact cells compared with cell debris at all doxorubicin concentrations tested. (**b**) In both passages tested viable cells decrease in a dose-dependent manner. Cells at low passage were more resistant to higher concentrations of doxorubicin (significance bars), except for 1000 nM. (**c**) The amount of apoptotic cells rises in parallel to the concentration of doxorubicin. Doxorubicin-induced Apoptosis was higher in cells at high passages in almost all concentrations tested (significance bars). (**d**) Incubation with doxorubicin was not able to give a significant increase of dead cells. Data are displayed as mean (SD); *p < 0.05, **p < 0.01, and ***p < 0.001.

After exposure to different concentrations of doxorubicin during 72 h. The amount of intact cells at low and high passage decreased from 91.1% (SD, 0.4%), and 86.5% (SD, 4.3%) in non-treated cells to 55.8% (SD, 3.1%), and 58.1% (SD, 2.1%) at the highest concentration of doxorubicin (2000 nM). In contrast, the amount of cell debris was consistently low in non-treated cells and increased proportionally to the concentration of doxorubicin, ranging from 8.2% (SD, 0.5%), and 13.5% (SD, 4.3%) in untreated cells at low and high passage to 43.3% (SD, 3.7%), and 41.9% (SD, 2.6%) at 2000 nM of doxorubicin, respectively. Response to doxorubicin—in terms of amount of intact cells and cellular debris—was not significantly different between low and high passages at any of the doxorubicin concentrations tested (Fig. 9a).

Intact cells were gated for viability on three different subpopulations as follows: viable, apoptotic, and dead cells. A significant dose-dependent reduction of viable cells at low and high passages starts after 72 h incubation with 50 nM of doxorubicin (*p* < 0.0001, and *p* = 0.0003, respectively). Viable cells at low and high passage decreased from 96.9% (SD, 0.4%), and 97.2% (SD, 0.2%) in non-treated cells to 54.6% (SD, 10.6%), and 30.9% (SD, 0.7%) at 2000 nM doxorubicin. The number of living cells was significantly higher in low-passaged cells at 50 nM, 100 nM, 200 nM, 500 nM and 2000 nM doxorubicin, more details are shown in Fig. 9b.

Doxorubicin induces dose-dependent apoptosis in TiHo-0906 cells at low and high passages, ranging from 5.2% (SD, 1.4%), and 4.6% (SD, 0.7%) at 1 nM doxorubicin to 45.1% (SD, 10.6%), and 65.9% (SD, 1.6%) at 2000 nM doxorubicin. The amount of apoptotic cells was significantly higher in high-passaged cells at all doxorubicin concentrations up to 50 nM (Fig. 9c). Surprisingly, incubation with doxorubicin reduced the amount of viable cells while increasing that of apoptotic cells but was not able to significantly increase the amount of dead cells. Nonetheless, part of the cell debris excluded from viability gating could represent fragments of dead cells. Incubation with 50 nM doxorubicin induced the highest amount of dead cells observed (5.9%; SD, 1.6%). Comparing low and high passages, the amount of dead cells was only higher in cells at high passage incubated with 2000 nM doxorubicin (Fig. 9d).

## Discussion

Epithelial to mesenchymal transition can be observed *in vitro* and has been immunohistochemically portrayed in mammary cancer in dogs and humans^48–50^. The importance of EMT on FMCs has not been evaluated, and to the extent of our knowledge, EMT-associated genomic aberrations have not been documented in an FMC cell line. TiHo-0906 cells displayed a combined epithelial/mesenchymal phenotype and a high expression of EMT-related markers *HMGA2* (qPCR) and *CD44* (qPCR and immunohistochemistry). Moreover, the cell line was characterized by stable proliferation and migration activity during subculturing, and specific CNGs in FCAs B4 and F2 harbouring known EMT-associated genes as *VIM*, *ZEB1*, *KRAS*, *HMGA2*, *ESRP1*, *MTDH*, *YWHAZ*, and *MYC*^7,22,23,36,51–54^. The TiHo-0906 cell line represents a unique cellular model, probably derived from a poorly differentiated cellular subpopulation encompassed in the original tumour.

The cell line was derived from a tumour composed of malignant tubular-epithelial cells, malignant spindle cells, and small anaplastic areas with polygonal cells. Immunohistochemically, tubuli-forming areas showed a higher affinity for CKs while spindle areas were characterized by a mesenchymal profile. Interestingly, polygonal cells in anaplastic areas and TiHo-0906 cells co-expressed CKs and mesenchymal markers. In human breast cancer, this is associated with poor prognosis^27,55,56^, in canine and human cellular models, with malignancy^38,55^.

TiHo-0906 cells displayed an increasing genetic instability during sub-cultivation. Genomic aberrations observed in the cell line were not present in the tumour. This may be related to the fact that DNA isolated from the tumour represents a genomic mixture of all cellular subpopulations included in the sample^19^, whereas the cultivated cells show clonal selection favouring a genomic shift. TiHo-0906 cells protein expression profile was more similar to that of the anaplastic polygonal cells in the tumour. This sub-population was, in fact, the least frequent of the three main cellular components of the tumour. However, considering that during the process of cell line establishment, cells with the higher rate of proliferation are likely to have a better survival chance in culture, the polygonal tumour cells may represent the cellular subpopulation with the faster growth rate and the higher amount of somatic aberrations. These findings also highlight the importance of cellular models as unique tools to study distinct aspects of the oncologic process specifically.

The tumour described in this study may represent a different histological subtype “FMCs with anaplastic and malignant spindle cells” from those included in the last WHO classification^2^. Matsuda *et al*.^57^ reported a “tubulopapillary carcinoma” with spindle cells (CKs+, and SMA+) in a cat^57^. In contrast, the spindle-cell component in the tumour in this study was negative for CKs but positive for SMA. Paniago *et al*.^58^ reported a “feline mammary carcinosarcoma” mainly composed of malignant pleomorphic cells, but no spindle cells were described^58^. Similar to the survival of the patient described in this study (three months), the animals reported by Matsuda *et al*.^57^ and Paniago *et al*.^58^ developed pulmonary metastasis and died three and two months after diagnosis, respectively^57,58^.

Similar to the tumour described in this study, spindle-hMBCs are characterized by a dominant fibromatosis-like pattern with epithelial cells and limited regions of polygonal cells arranged in clumps or cords^59,60^. Immunohistochemically, spindle-hMBCs coexpress CKs (cytokeratin 8/18 [CK8/18], pan-CK, cytokeratin 14 [CK14], and CK5/6) and mesenchymal markers (SMA and Vim)^59,60^. Spindle cells in these tumours are malignant and display a variable reaction to CKs^61–63^. Therefore, a wide spectrum keratin panel (including pan-CK) may be useful for differentiating spindle-hMBCs from other spindle-cell lesions^60^. In the tumour described in this study, the spindle cells expressed SMA, CALP and Vim but lacked CKs including pan-CK, which according to the aforementioned would suggest a more likely mesenchymal phenotype. Since the tumour and derived cell line were enriched with EMT traits, and both, polygonal and spindle cells in the tumour expressed CD44, it appears reasonable to consider that both components may have arisen from EMT. However, we cannot completely rule out the presence of some non-neoplastic reactive mesenchymal cells (i.e. myofibroblasts) within the spindle component of the tumour. Considering the presence of both epithelial and mesenchymal components, carcinosarcoma would represent another possible differential diagnosis^64^. In dogs, mammary carcinosarcomas (commonly carcinoma and osteosarcoma) are described to express CK8/18 and Vim, but not CALP, p63 and CK14^64^. Although this expression profile has not been demonstrated in cats so far, a mammary gland carcinosarcoma is very unlikely in the present case on the basis of histological and immunohistochemical findings. Nonetheless, whether the spindle cells were originated from EMT or represented an additional neoplastic mesenchymal population remains at this point unanswered. In summary, the tumour described in this study shows some similarities with the spindle-hMBC, and it also resembles the FMC cases described by Matsuda *et al*. and Paniago *et al*.^57,58^.

HMGA2 induces EMT^7,22–26,65,66^. In this study, we compared the absolute *HMGA2* qPCR-based expression of TiHo-0906 cells with a well-known reference tissue (testis) characterized by high expression of *HMGA2*^67^. The quantitative expression of *HMGA2* was significantly higher in TiHo-0906 cells when compared to the reference tissue. These findings are concordant with the CNGs observed in chromosome B4 harbouring *HMGA2*, which in combination with the characteristic fibroblastoid-shape and the highly migratory ability (demonstrated by *in vitro* scratching assay) also reveal a profoundly altered mesenchymal gene expression program; consistent with the combined epithelial/mesenchymal phenotype observed.

hMBCs coexpress EMT- and CSCs-features^12^. Accordingly, these tumours may originate directly from epithelial cells that have undergone EMT and have therefore acquired CSCs properties, or from pre-existing CSCs expressing EMT-associated markers^8^. In FMC and human breast cancer cell lines, CSCs express high levels of CD133, CD44 and low (or none) CD24^30,68,69^. In this study, the immunohistochemical expression of CD24 and CD133 was not assessed due to the unavailability of the specific antibodies. However, the fact that only some TiHo-0906 cells expressed p63 when the original tumour was negative, may suggest the presence of some cells with stem cells features which may have acquired a myoepithelial phenotype during sub-cultivation as previously described in a human mammary gland derived cell line^70^. All cell types in the tumour expressed CD44, and there was a specific intense membrane staining for CD44 on almost all TiHo-0906 cells. The quantitative gene expression of *CD44* was also significantly higher in the cell line than in the reference tissue. These findings together with the comparative expression profile and *HMGA2* expression also suggest that the cell line was originated from the anaplastic polygonal cells in the tumour.

Several well-described breast cancer- and EMT-associated genes were affected by CNVs in the genome of the cell line. As reported by Zhao *et al*.^36^ in a study exploring the relationship between EMT-implicated gene expression and CNVs in multiple cancer types in humans^36^; we observed that EMT-associated genes were more frequently affected by CNGs than by CNLs. As was mentioned before, most of the EMT-related genes affected by CNVs were located in amplified regions of FCAs B4 and F2. Affected regions of chromosome F2 harboured four of the most commonly EMT-associated genes reported by Zhao *et al*.^36^; this includes *ESRP1*, *MTDH*, *YWHAZ*, and *MYC*^36^. Among those, *ESRP1* regulates CD44 alternative splicing producing a more malignant isoform (CD44v), promoting invasiveness and distant metastasis in human breast CSCs^71^. TiHo-0906 cells overexpress CD44 and displayed CNGs in chromosome F2 harbouring *ESRP1* gene. Therefore, TiHo-0906 cell line might be a suitable model for the study of *ESRP1* and CD44 as possible therapeutic targets for FMC.

CNGs in chromosome F2 observed in the TiHo-0906 cells resemble one of the most common DNA aberrations reported in human breast cancer cell lines—CNGs at the long arm of human chromosome 8 (HSA 8q)—^35,72^. When comparing the human and feline karyotypes using fluorescence *in situ* hybridization (FISH), the entire HSA 8q is hybridized by the paint from FCA F2^73,74^. CNGs in chromosome F2 observed in the cell line also included some proposed breast cancer-related genes like *EIF3E*, *PKHD1L1*, *CSMD3*, *ZHX2*, *SAMD12*, *EXT1*, *MRPL13*, *MTBP*, *SNTB1*, *RNF139*, and *TATDN1*^35^. These findings highlight the importance of FCA F2 in FMC development and the close evolutionary relationship with the homologous disease in humans. Additionally, TiHo-0906 cells at low and high passage also displayed CNGs in regions harbouring other known breast cancer-related genes like *FOXO3* and *MYB* (FCA B2), *AKT1* (FCA B3), and *GATA-3*, *CCND2*, *CDK4* and *PFDN5* (FCA B4).

CNLs were less common than CNGs. Most CNLs observed affected low or high passages of the cell line, but few of them affected both. For instance, CNLs in chromosome D2, and E3 harbouring *PTEN*, and *RAC1*, respectively; were preserved during prolonged subculturing. In contrast, CNLs in chromosome B1 were only observed in low-passaged cells. This chromosomic region harbours the *CSMD1*, *AGPAT5*, *TUSC3*, *DLC1*, *CLDN23*, and *MFHAS1* cancer-related genes. This aberration corresponds with the most common CNL in human breast cancer cell lines, located at the short arm of human chromosome 8 (HSA 8p)^35^. Using FISH, feline chromosome B1 labels the entire HSA 8p^73,74^. Additional CNLs affecting known breast cancer-, hMBCs-, and EMT-related genes were only detected after prolonged subculturing; including, FCA A1 containing *TERT*, *BRCA2*, *RB1*, and *FOXO1* genes; chromosome A2 harbouring *EGFR*, *TWIST*, *MLL2*, and *AHR*; chromosomes B2, B3, and B4 harbouring *CCND3*, *FOXA1*, and *MDM2*, respectively; and chromosome E1 harbouring *NF1*, *PPM1D*, and *KSR1*.

TiHo-0906 proliferation and migration activity remained comparable during subculturing. Growth curves and doubling times (28.9 h at low passage and 27.4 h at high passage) revealed a high proliferation similar to those reported for previously characterized FMC cell lines^39,75–77^. Migration activity was assessed by *in vitro* scratch assay revealing a shorter time to complete wound closure (approximately 14 h for both, early and high passages) than that previously reported in a recently characterised FMC cell line (approximately 24 h)^39^.

After doxorubicin treatment, we observed a significant dose-dependent reduction of viable cells with active metabolism only after 72 h incubation with 100 nM of doxorubicin at low and high passages. These data may suggest a higher doxorubicin resistance of TiHo-0906 cells to that reported in an FMC cell line^78^. Nonetheless, methodological differences in viability assessment and exposure time to doxorubicin make comparison difficult. Resistance to doxorubicin, in terms of metabolic activity remained comparable during subculturing. However, flow cytometric assessment of doxorubicin effects on TiHo-0906 cells revealed a significant increase of apoptotic cells in high-passaged cells when compared to low-passaged cells at all doxorubicin concentrations up to 50 nM (except for 1000 nM). These findings are consistent with the observed two-fold reduction of the IC50 during sub-cultivation, suggesting a reduction in chemoresistance during prolonged subculturing. Similar findings have been reported for different cellular models in humans^79^. However, the specific mechanisms underlying this process are still not clear. To the best of our knowledge, resistance to doxorubicin has not been previously assessed by flow cytometry in FMC-derived cell lines. In this case, we observed a dose-dependent reduction of vital cells with simultaneous increasing of apoptotic cells.

Some immunohistochemical studies in veterinary medicine support the EMT hypothesis to explain the origin of the spindle-cell component on canine mammary metaplastic tumours^48,49^. There is also evidence suggesting a direct CSCs origin in cell lines derived from canine mammary spindle-cell tumours and sarcomas^80^. Although these two theories have been considered separately, a growing body of literature in human medicine suggests a link between them^8,13,14,50^. *In vitro* culture models are pivotal to understanding the role of EMT in the oncogenic process of FMC, determining the presence of possible subtypes and developing of new therapeutic approaches. However, the number of established FMC cell lines is still small^30,39,75–78,81–83^.

In summary, the present study demonstrated that TiHo-0906 cells co-expressed epithelial and mesenchymal features, and some EMT markers like *HMGA2* and *CD44*. Additionally, TiHo-0906 cells are characterized by stable metabolic activity, proliferation rate, and ability to migrate during prolonged subculturing. Furthermore, our results indicate that specific CNVs observed in the cell line harbour a considerable amount of genes implicated in breast cancer development and EMT. CNGs in chromosomes B4 and F2 may be essential for EMT activation in the cat genome and closely resemble some of the typical genomic aberrations observed in human breast cancer. Nonetheless, further studies are necessary to determine the importance of these genomic aberrations in FMCs. The TiHo-0906 cell line represents a unique model consistently displaying EMT-associated traits at baseline. Considering the poor prognosis of affected animals, further studies using *in vitro* models that closely resemble the uniqueness of the original tumours are of major value for the development of potential therapeutic approaches.

## Methods

### Primary tumour tissue

The TiHo-0906 cell line was obtained from the primary lesion of a 13-year-old female intact British Shorthair cat diagnosed with spontaneous FMC at clinical stage three according to McNeill *et al*.^84^. The cat was admitted to the Small Animal Clinic at the University of Veterinary Medicine Hannover and underwent unilateral chain mastectomy. No chemotherapy was performed and the cat was euthanised three months after surgery due to pulmonary metastasis. Tissue samples were obtained during the medically necessary surgery, after signed informed owner consent. Consequently, an ethical approval was not required (German Animal Welfare Act, § 7).

### Cell culture establishment and maintenance

Immediately after surgery, a tumour tissue sample was placed in a tube with Hank’s Balanced Salt Solution (Gibco) and kept overnight at 4 °C. The remainder of the surgical specimen was fixed in 10% neutral-buffered formalin and prepared for histopathological examination. The selected tumour tissue was minced into small pieces and digested with 4 mL 0.26% collagenase (SERVA) for 3 h at 37 °C with constant stirring. The digested cells were transferred into a sterile 25 cm^2^ cell culture flask containing 5 mL medium 199 (Gibco) supplemented with 20%foetall bovine serum (FBS, Biochrom) and 2% penicillin/streptomycin (Biochrom). The culture was incubated at 37 °C in a humidified atmosphere of 5% CO_2_ and split at confluency once or twice a week utilizing 1 mL TrypLE^TM^ (Gibco) as dissociation reagent. Cryogenic preservation was performed once a week at 90% confluence. Cells were dispersed in TrypLE^TM^ and resuspended in growth medium (medium 199 supplemented with 10% FBS, and 2% penicillin-streptomycin). The cell suspension was centrifuged at 1000 g for 10 min, the supernatant was discarded and the cell pellet was resuspended in 1 mL freezing medium (medium 199 supplemented with 20% FBS, 2% penicillin-streptomycin and 10% DMSO [AppliChem]) and transferred into cryogenic storage vials. Cryogenic vials were accommodated in a freezing container (Mr. Frosty, Nalgene), and transferred to a −80 °C freezer, after 24 h the vials were stored in a −150 °C freezer or in a liquid nitrogen storage vessel. TiHo-0906 was considered established when overcoming the passage 30^th^ (P30).

All experiments described below were carried out in triplicate; those involving cells were performed using TiHo-0906 cells, or cell-pellets (5 × 10^6^ cells) at low and high passage (P7–P10, and P76–P80, respectively). For pellets preparation, cells were detached with 1 mL TrypLE^TM^, rinsed with PBS, counted with a PC-based Cellometer (Auto T4, Nexcelom Bioscience), and centrifuged at 1000 g for 10 min. For DNA and RNA isolation, pellets were stored at −80 °C. For cytology and immunohistochemistry, pellets were fixated in 10% neutral-buffered formalin and stored at room temperature.

### Histopathology and cytology

After formalin fixation, paraffin sections (4 µm) of the original tumour were stained with H&E for histopathologic evaluation. The samples were examined under light microscopy and the morphological diagnosis was performed following the WHO classification^2^. Histological grading of the tumour was performed according to the method described by Elston and Ellis 1991 and Castagnaro *et al*.^85,86^. The cytomorphologic features of the cell line during long-term cultivation were examined microscopically based on H&E stained formalin-fixed, paraffin-embedded cell pellets at low (P7) and high (P80) passages.

### Immunohistochemistry

Immunophenotyping by the avidin-biotin method was performed in the original tumour and cell pellets of TiHo-0906 (low [P7], and high [P80] passages) as described elsewhere^46^ using the following markers (details see Table 6): E-cad, CK8/18, pan-CK, CK14, CK5/6, p63, SMA, CALP, Vim, HMGA2, CD44, ER, PR, HER-2, COX-2, p53, CLDN-2 and proliferation marker Ki-67. Positive and negative controls used for immunohistochemistry are listed in the Supplementary Table 1. For negative controls, the specific primary antibody was replaced by either an isotype control antibody or normal serum. For positive controls, only tissue samples were used that were sent to the department of pathology for diagnostic purposes not related to the present study. No animal was killed for the generation of positive controls.

**Table 6:** Antibodies and evaluation methods used in this study.

Antibody	Type	Clone	Company	References
E-cad	mouse anti-human*	36/E-cad	BD Biosciences	^96, 97^
CK8/18	mouse anti-human*	5D3	Novocastra	^96, 97^
pan-CK	mouse anti-human*	AE1&AE3	Dako	^96, 97^
CK14	rabbit anti-human**	—	Thermo Fischer Scientific	^96, 97^
CK5/6	mouse anti-human*	D5/16B4	Dako	^96, 97^
p63	mouse anti-human*	4A4	Biologo	^96, 97^
SMA	mouse anti-human*	1A4	Dako	^96, 97^
CALP	mouse anti-human*	CALP	Dako	^96, 97^
Vim	mouse anti-human*	V9	Dako	^96, 97^
HMGA2	rabbit anti-human**	HMGA2	Lifespan Biosciences	^98^
CD44	Rat anti-mouse CD44*	IM7	Bio-Rad Laboratories	^30, 82^
ER	mouse anti-human*	6F11	AbD Serotec	^96, 97, 99^
PR	mouse anti-human*	10A9	Immunotech	^96, 97, 99^
HER-2	mouse anti-human*	CB11	Novocastra	^94, 95^
COX-2	goat polyclonal IgG	—	Santa Cruz	^93^
p53	mouse anti-human*	DO-1	AbD Serotec	^100^
CLDN-2	mouse anti-human*	12H12	Thermo Fischer Scientific	^42^
Ki-67	mouse anti-human*	MIB-1	Dako	^101^

*Monoclonal Antibody, **Polyclonal Antibody.

### Copy number variation analysis

Four 10-µm-thick sections were sliced from the tumour FFPE-block, using a microtome (pfm Slide 2003). Afterwards, the sections were deparaffinized and the DNA was isolated with the AllPrep DNA/RNA FFPE kit (QIAGEN) according to manufacturer’s instructions. Before DNA isolation, 4 × 10^6^ TiHo-0906 cells (P7 and P76) were homogenized using QIAshredder^TM^ columns (QIAGEN). DNA isolation was performed with the AllPrep DNA/RNA Mini Kit (QIAGEN) following the manufacturer’s protocol. DNA yields and purity (260/280 ratio) were quantified with the Synergy 2 plate-reader (BioTek).

Starting from the DNA a sequencing library for each sample was prepared using the NEBNext® Ultra^TM^ II DNA Library Preparation Kit for Illumina® (New England Biolabs). Before library preparation, the DNA was sheared by ultrasound to an approximate size of 500 bp. An input of 500 ng DNA was used for each library. Sequencing was conducted on an Illumina NextSeq500 according to manufacturer’s instructions. The program CNV-seq (http://bmcbioinformatics.biomedcentral.com/articles/10.1186/1471-2105-10-80) was used for copy-number analyses; using feline healthy mammary tissue as normal reference control and a fixed window size of 1,000,000 bp. Control-tissue was collected from a recently euthanized intact female cat with no pathologies of the mammary gland. The raw results (log2ratios) were smoothed using a circular binary segmentation algorithm implemented in the program “Copynumber” (http://bioconductor.org/packages/release/bioc/html/copynumber.html). Based on the results of the normal reference control, regions with log2-copy-number ratios of >0.25 or <−0.25 were scored as significantly aberrant. Additionally, some bins at telomeres were removed. All bins with smoothed log2-copy-number ratios of >0.25 were scored as amplified (CNGs) and all bins with <−0.25 were scored as deleted (CNLs).

Structural rearrangements (CNGs, and CNLs) observed were compared with those reported in human breast cancers^33,34^, and were complemented by important EMT-related CNVs reported in multiple human cancer types^36^, specific hMBCs-related CNVs^7,32^, breast cancer cell lines-related CNVs^35^, and additional cancer-associated genes reported on FMCs.

### Real-time PCR expression analyses of *HMGA2* and *CD44*

HMGA2 expression is nearly undetectable in adult tissues except for meiotic and post-meiotic cells, in which its expression is high^67,87^. Consequently, we used feline testis as reference tissue. CD44 is overexpressed in metastatic mammary cancer tissues and CSCs, while its expression in the normal mammary gland is very low^88–90^. Accordingly, we used healthy mammary tissue as reference tissue. Testicular tissue was collected from a healthy tomcat during elective orchiectomy and mammary tissue from a recently euthanized (for medical reasons) intact female cat with no pathologies of the mammary gland.

Total RNA was isolated from TiHo-0906 cells (P8 and P80), fresh-frozen feline testicular tissue (*HMGA2* reference) and non-neoplastic feline mammary tissue (*CD44* reference) using the RNeasy Mini Kit (QIAGEN) according to manufacturer’s protocol. The RNA yield and purity (260/280 ratio) were determined using a plate reader (Synergy 2, BioTek). The RNA was stored at −80 °C until use.

*HMGA2* and *CD44* mRNA levels were measured using absolute quantification, relative to amplicon-specific standard curves. *HMGA2* standard curve sequence and amplification primers were based on a custom canine assay^91^ and adjusted to the feline sequence. The feline *HMGA2* sequence was obtained by aligning the human exon 2, 3 and 4 sequences (NM_001300918.1) against cat chromosome B4. The feline standard sequence was (5′–3′): *HMGA2* cat standard: AGAGTCCCTCCAAAGCAGCTCAAAAGAAAGCAGAAGCCACTGGAGAAAAACGGCCAAGAGGCAGACCCAGGAAATGGCCA. The primers were (5′–3′): *HMGA2* cat fw: AGTCCCTCCAAAGCAGCTCAAAAG and HMGA2 cat rv: GCCATTTCCTGGGTCTGCCTC. *CD44* standard curve sequence and amplification primers were based on Ensembl-ID ENSFCAT00000005889.4, lying on the exon 3 and 4 border. *CD44* cat standard (5′–3′): TACATCGGTCACACACCTGCCCAATGCCTTTGAAGGACCAATTACCATAACCATTGTTAACCGTGATGGCACCCGCTATA. The primers were (5′–3′): *CD44* cat fw: CATCGGTCACACACCTGCCC and CD44 cat rv: TAGCGGGTGCCATCACGGTT.

Real-time PCR was carried out using the Mastercycler® ep realplex Real-Time PCR System (Eppendorf). The qPCR reactions were performed using the QuantiTect SYBR® Green RT-PCR Kit (QIAGEN) in a total reaction volume of 20 µL and a primer concentration of 1 µL of 10 µM stock, for each primer. 100 ng of isolated total RNA were used per sample to measure mRNA copy numbers. The standard curves resulted from seven dilution steps, ranging from 10^2^ to 10^8^ copies. PCR conditions were as follows: 50 °C for 30 min cDNA synthesis, 95 °C for 15 min initial denaturation, 40 cycles at 94 °C for 15 s, 60 °C for 30 s and 72 °C for 30 s, completed by a melting curve analysis. All samples including the dilutions of the standard curve were measured in triplicate.

### Growth behaviour and migration activity

TiHo-0906 cells proliferation was measured via Cell Proliferation ELISA, BrdU (colorimetric) Assay (Roche). TiHo-0906 cells (7.5 × 10^3^ cells/well) at low (P7) and high (P76) passages were seeded in 96-well plates in octuplets and incubated at 37 °C in a humidified atmosphere of 5% CO_2_. BrdU was added after 24 h, 48 h, 72 h and 86 h and incubated for 18 h each time. BrdU absorbance values at 370 nm (reference wavelength 492 nm) were recorded using a plate reader (Synergy 2, BioTek).

For calculating doubling time and growth curves, TiHo-0906 cells (P10 and P79) were seeded (7.5 × 10^5^ cells/well) in 6-well plates in triplicates and incubated at 37 °C in a humidified atmosphere of 5% CO_2_ for four days. Every 24 h, three replicative wells were dissociated with 1 mL TrypLE^TM^ and the cells were counted using a PC-based Cellometer Auto-T4 (Nexcelcom Bioscience). The growth curves were established, and the doubling times were calculated using the “Doubling Time” online tool (http://doubling-time.com/compute.php).

TiHo-0906 cells migration ability was evaluated using the *in vitro* wound-scratching assay as previously reported^39^. Cells at low (P7) and high (P76) passages were cultured in 6 well culture plates in triplicates, once the cell confluence reached 100%, the growth medium was removed, the cell monolayer was gently scratched using a 200 µL pipette tip and rinsed with PBS, and the growth medium was replaced. Afterwards, cells were photographed every 10 min during 24 h using a live cell imaging microscope (DMI 6000 B, Leica Microsystems). Time to wound closure was defined as the time at which the wound was completely filled with cells.

### Sensitivity to doxorubicin

The anti-proliferative effects of doxorubicin on TiHo-0906 cells were evaluated by assessing the cells metabolic activity with the CellTiter 96® MTS Proliferation Assay (Promega). Cells at low (P7) and high (P79) passages were seeded in 96-well plates (7.5 × 10^3^ cells/well) and incubated for 18 h. The medium was aspirated and the cells were treated with different dilutions of doxorubicin (1 nM, 10 nM, 100 nM, 200 nM, 1000 nM, and 2000 nM [200 μL/well]). These concentrations of doxorubicin were below the range of the *in vivo* maximum plasma concentration in cats treated with clinically relevant dosages^92^. Each concentration was added in quadruplets, every plate contained a control of cells in growth medium and a negative control of the medium. After 72 h of incubation, the medium was changed, and 20 μL of the CellTiter 96® solution were added to each well. The plates were incubated at 37 °C in a humidified atmosphere of 5% CO_2_ for 2 h and the absorbance values were recorded (reference wavelength 492 nm) and normalized to the medium negative control using a plate reader (Synergy 2, BioTek). The inhibitory activity of doxorubicin on TiHo-0906 cells at low and high passage was assessed by dose-response curves and IC50 using GraphPad Prism 3.0 (GraphPad Software Inc., San Diego, CA, USA), as calculated from the data on metabolic activity.

### Apoptosis induced by doxorubicin

TiHo-0906 cells (P7 and P77) were seeded in 6-well plates (2 × 10^5^ cells/well) and incubated for 18 h at 37 °C in a humidified atmosphere of 5% CO_2_. Afterwards, the cells were exposed to different dilutions of doxorubicin (1 nM, 10 nM, 50 nM, 100 nM, 200 nM, 500 nM, 1000 nM, and 2000 nM [3 mL/well]) and then re-incubated for 72 h. After this period, media containing non-adherent and dead cells were collected. Adherent cells were dissociated with 1 mL TrypLE^TM^ and centrifuged (1000 rpm for 6 min) together with the previously collected media. The supernatant was discarded, and cell pellets were resuspended in 500 μL of 1X Binding Buffer. Cell suspensions were incubated (10 min at room temperature in the dark) with 5 μL Annexin V-FITC and 1 μL SYTOX Green Dye (Annexin V-FITC Detection Kit plus, PromoCell). Flow cytometry was performed using a MACSQuant® Analyzer 10 (Miltenyl Biotec). Dead cells treated with 1 mL 0.2% Triton^TM^ X-100 (Sigma-Aldrich) and non-treated viable cells were used to set the gates. Annexin V-FITC and SYTOX Green Dye were measured in the FL-1 channel, and results were analysed with FlowJo Version 7.6.5 (FlowJo, Ashland, OR, USA). For data analysis, cell debris on the left-side of the plot were set as negative gate. Afterwards, intact cells were gated for viability on three different populations: viable, apoptotic, and dead.

### Statistical analysis

All statistical analyses were performed using SAS software 7.1 (SAS Institute Inc., Cary, NC, USA); the significance threshold was set at *p* ≤ 0.05. Data distribution was tested using Shapiro-Wilk test. Statistical analysis of BrdU cell proliferation test, growth curves, and real-time PCR expression analyses of *HMGA2* and *CD44* were performed using a 2-tailed Mann-Whitney-U test. Times to *in vitro* wound-scratch closure were analysed by two-tailed t-test. Results from MTS cell proliferation test and flow cytometry were evaluated using Wilcoxon’s two-sample test.

## Electronic supplementary material

Supplementary table 1
