# Supplementary material for: TiHo-0906: a new feline mammary cancer cell line with molecular, morphological, and immunocytological characteristics of epithelial to mesenchymal transition
Source: Sci Rep. 2018 Sep 5;8:13231. doi: 10.1038/s41598-018-31682-1 (PMC6125410; doi:10.1038/s41598-018-31682-1)
Supplement: Supplementary file 1 — Supplementary table 1 [file 41598_2018_31682_MOESM1_ESM.docx]

**TiHo-0906: a new feline mammary cancer cell line with molecular, morphological, and immunocytological characteristics of epithelial to mesenchymal transition**

José Luis Granados-Soler, Johannes Junginger, Marion Hewicker-Trautwein, Kirsten Bornemann-Kolatzki, Julia Beck, Bertram Brenig, Daniela Betz, Jan Torben Schille, Hugo Murua Escobar, Ingo Nolte

**Supplementary Information**

| **marker** | **negative control** | **positive control** |
| --- | --- | --- |
| vimentin | IgG1 | feline skin |
| pan-CK | IgG1 | feline skin |
| CK8/18 | IgG1 | feline normal mammary gland |
| CK14 | rabbit serum | feline skin |
| CK5/6 | IgG1 | feline skin |
| SMA | IgG2a | feline urinary bladder |
| calponin | IgG1 | feline normal mammary gland |
| p63 | IgG2a | feline normal mammary gland |
| E-cadherin | IgG2a | feline skin |
| Ki67 | IgG1 | feline normal small intestine |
| p53 | IgG2a | feline transitional cell carcinoma |
| COX-2 | rabbit serum | feline normal lymph node, feline transitional cell carcinoma |
| HER-2/c-erbB2 | IgG1 | human mammary tissue and pellets from cell lines  overexpressing HER-2*, feline mammary carcinoma |
| progesteron receptor | IgG2a | feline normal mammary gland |
| estrogen receptor | IgG1 | feline normal mammary gland |
| claudin-2 | IgG2b | feline and canine normal mammary gland |
| HMGA2 | rabbit serum | Feline and canine neonatal tissue |
| CD44 | rat serum | feline lymph node |
| *kindly provided by Prof. Dr. H.-H. Kreipe, Department of Pathology, Hannover Medical School | | |

**Supplementary table 1.** Antibodies and corresponding negative and positive controls used in this study.
